# Puerarin protects against sepsis-induced myocardial injury through AMPK-mediated ferroptosis signaling

**DOI:** 10.18632/aging.204033

**Published:** 2022-04-28

**Authors:** Bin Zhou, Jing Zhang, Yixuan Chen, Yang Liu, Xiaoyi Tang, Panpan Xia, Peng Yu, Shuchun Yu

**Affiliations:** 1 Department of Anesthesiology, The Second Affiliated Hospital of Nanchang University, Nanchang 330006, Jiangxi, China; 2 The Second Clinical Medical College of Nanchang University, The Second Affiliated Hospital of Nanchang University, Nanchang 330006, Jiangxi, China; 3 Department of Metabolism and Endocrinology, The Second Affiliated Hospital of Nanchang University, Nanchang 330006, Jiangxi, China

**Keywords:** myocardial injury, Puerarin, LPS, AMPK, ferroptosis

## Abstract

Objective: Research suggests that Puerarin may protect against sepsis-induced myocardial damage. However, the mechanisms responsible for Puerarin’s cardioprotective effect remain largely unclear. In this study, our objective is to investigate the role of Puerarin-induced AMPK-mediated ferroptosis signaling in protecting myocardial injury.

Methods: 48 male Sprague-Dawley rats were randomly divided into four groups: control group, LPS group, LPS + Pue group, LPS + Pue + Era (Erastin, ferroptosis activator) group, or LPS + Pue + CC (compound C, AMPK inhibitor) group. During the experiment, cardiac systolic function indexes and myocardial histopathological changes were monitored. The serum levels of myocardial injury marker enzyme, inflammatory response related marker enzyme, and oxidative stress related-marker enzyme were measured with ELISA. Apoptotic cardiomyocytes, the iron content in myocardial tissue, apoptosis-related proteins, AMPK, and ferroptosis-related proteins were determined.

Results: Puerarin inhibited the myocardial injury induced by LPS. The cardioprotective effects of Puerarin decreased after adding ferroptosis-activating compound Erastin. The protein expression levels of GPX4 and ferritin were down-regulated, whereas ACSL4, TFR, and heart iron content were up-regulated in LPS + Pue + Era group compared with LPS+Pue group. A significant difference was identified between LPS + Pue + Era group and LPS + Pue group in P-AMPK and T-AMPK levels. Meanwhile, after providing CC, P-AMPK/T-AMPK was significantly reduced, the protein expression levels of GPX4 and ferritin were down-regulated. ACSL4, TFR, and the heart iron content were up-regulated in LPS + Pue + CC group compared to LPS + Pue group.

Conclusions: Puerarin protected against sepsis-induced myocardial injury, and AMPK-mediated ferroptosis signaling played a crucial role in its cardioprotective effect.

## INTRODUCTION

Sepsis is a chronic systemic inflammatory response syndrome (SIRS) that can lead to septic shock and multiple organ dysfunction syndrome (MODS) [1, 2]. According to statistics, sepsis accounts for 11% of all acute and critical illnesses in ICUs, and its incidence increases by 8% to 13% annually [3]. Over 18 million patients with severe sepsis each year, with a 30-70% mortality rate. In critically ill patients, sepsis is one of the leading causes of death [4]. Sepsis is most frequently caused by Gram-negative bacteria [5]. The Gram-negative bacterial cell wall is primarily composed of lipopolysaccharides composed of lipid A, core polysaccharides, and O-antigens [1]. The elicitor functions after the invasion, triggering the inflammatory response and resulting in multiple organ dysfunction, ultimately leading to MODS [5]. The heart is one of the vulnerable target organs of sepsis. Research has demonstrated that 40%-50% of patients with sepsis have a myocardial injury, manifesting as arrhythmia, hypotension, and heart failure, and 20% of patients have latent myocardial injury [6, 7]. In addition, sepsis-mediated myocardial dysfunction has been proposed as a contributing factor to hospital-acquired mortality [8]. Studies have confirmed that various drugs effectively improve sepsis-induced myocardial injuries, such as metformin and ulinastatin [9, 10]. In recent years, using antibiotics has been rampant, leading to the emergence of resistant bacteria, implying the urgent need for novel drugs and innovative treatments for sepsis.

Sepsis patients have long been treated with traditional Chinese herbal medicine, which has had good therapeutic outcomes. Research has revealed that traditional Chinese medicine monomers and gene knockout technology can improve LPS-induced multiple organ dysfunction, including heart, lung, brain, kidney, and other essential organs [11]. Both berberine and artesunate can protect against acute lung injury caused by LPS [12, 13]. Concurrently, curcumin can effectively improve LPS-induced acute kidney injury [14]. In addition, studies have indicated that emodin can reduce damage to brain tissue caused by LPS [15]. Puerarin is the main active ingredient in the traditional Chinese medicine Pueraria. With its anti-inflammatory, anti-oxidation, and other effects, Puerarin has been deployed to prevent and treat cardiovascular disease [16]. Recent research reveals that Puerarin can reduce the risk of myocardial injury caused by LPS, inhibit inflammation, and reduce oxidative stress [17–19]. Puerarin inhibits the inflammatory response by inhibiting TLR4/NF-κB/JNK signaling pathway, improving LPS-induced multiple organ injury [17]. Puerarin reduced the myocardial injury induced by LPS by inhibiting the expression of inflammatory cytokines IL-1β, TNF-α and apoptosis-related protein Bax in an *in vitro* experiment [18].

Additionally, Puerarin can improve LPS-induced myocardial contractile dysfunction, increasing LVESP in septic rats [19]. Further research is required to determine the specific mechanism Puerarin uses to reduce sepsis-induced myocardial injury. Several factors have been implicated in the etiology of septic myocardial damage, including inflammation, oxidative stress, myocardial energy metabolism disorders, and excessive activation of the renin-angiotensin system (RAS) [20, 21]. Several scholars have proposed that ferroptosis plays an essential role in LPS-induced myocardial damage [22]. Iron-dependent ferroptosis is a brand new cell death model regulated by lipid oxidation and depends on iron [23, 24]. Ferroptosis occurs predominantly because Erastin inhibits the entry of cystine into a cell, resulting in glutathione depletion and inactivation of GPX4.

As a result, a large amount of Fe^2+^ enters mitochondria, causing a large volume of ROS to be produced, resulting in cell death [22, 23]. Dexmedetomidine inhibits ferroptosis by reducing HO-1 expression and increasing GPX4 expression, thereby reducing LPS-induced myocardial damage [25]. Ferroptosis is believed to be regulated by an AMPK signaling pathway, and activation of this pathway inhibits ferroptosis [26]. It should be observed that Puerarin stimulates AMPK and Akt signaling pathways in myocardial tissues, improving various myocardial diseases, including pathological myocardial hypertrophy and myocardial hypoxia/reoxygenation injury [27, 28]. Puerarin can also prevent heart failure caused by pressure overload by reducing ferroptosis [29].

We hypothesize that Puerarin prevents ferroptosis by acting on AMPK signaling pathway, thus protecting myocardial damage and cardiac dysfunction caused by LPS. Several basic and clinical studies have demonstrated that extracts from traditional Chinese herbal medicine can help protect against various cardiovascular diseases. Once Puerarin enters the body, it can be absorbed and utilized by the human myocardium, protecting myocardial cells and improving the ability to contact them. To further elucidate Puerarin’s protective mechanisms against LPS-induced myocardial injury, we used an *in vivo* sepsis model with LPS administration and corresponding medications to investigate the protective mechanisms of Puerarin.

## RESULTS

### Puerarin attenuates LPS-induced myocardial injuries and cardiac dysfunction in rats

**Figure 1:**
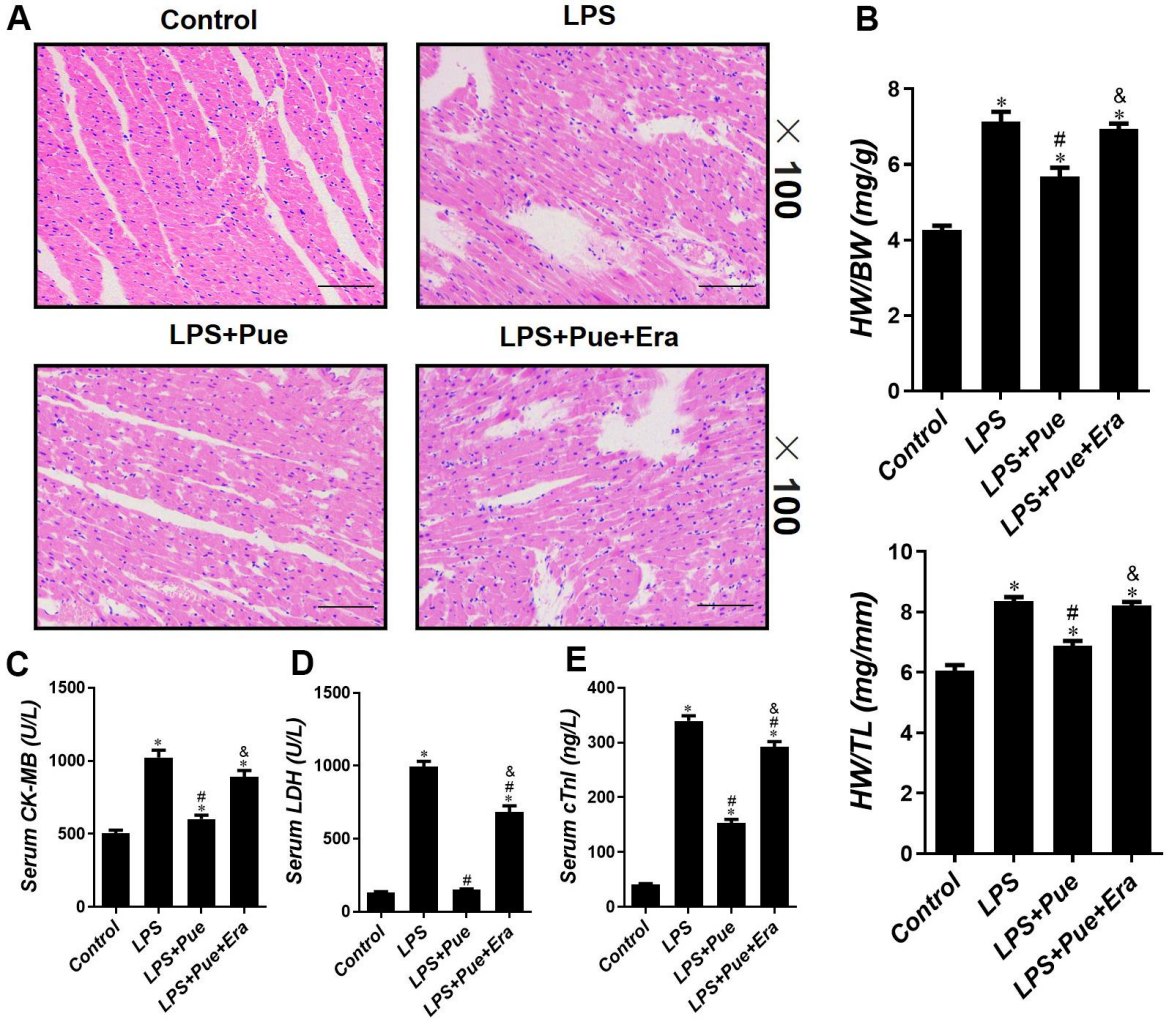
**Puerarin attenuates LPS-induced myocardial injuries in mice.** (**A**) He staining sections were used to observe the pathological changes of heart tissues. The ratio of HW/BW (**B**) and HW/TL (**B**) is used to reflect the degree of cardiac edema. The concentrations of (**C**) CK-MB, (**D**) LDH and (**E**) cTnI in serum of mice were determined by ELISA. Data represent the mean ± SD. *P < 0.05 vs Control group; #P < 0.05 vs. LPS group; &P < 0.05 vs. LPS+Pue group.

LPS has been demonstrated to cause myocardial damage. As expected, LPS group’s myocardium sections exhibited significant pathological changes compared to the control group, including myocardial fiber arrangement disorder, partial myocardial fiber rupture lysis, edema cardiomyocytes, dilated intercellular space, and numerous inflammatory infiltrating cells (Figure 1A). Compared to the control group, the ratios of HW/BW and HW/TL in LPS group increased significantly (p < 0.05) (Figure 1B). On the other hand, Pue significantly reduced pathological changes (Figure 1A). Compared to the LPS group, the ratios of HW/BW and HW/TL were significantly (p < 0.05) decreased in LPS+Pue group (Figure 1B). In contrast to LPS group, no significant pathological changes were observed in rats treated with Pue + Era (Figure 1A). Similarly, there was no significant difference in myocardial weight between the two groups (p > 0.05).

**Figure 2:**
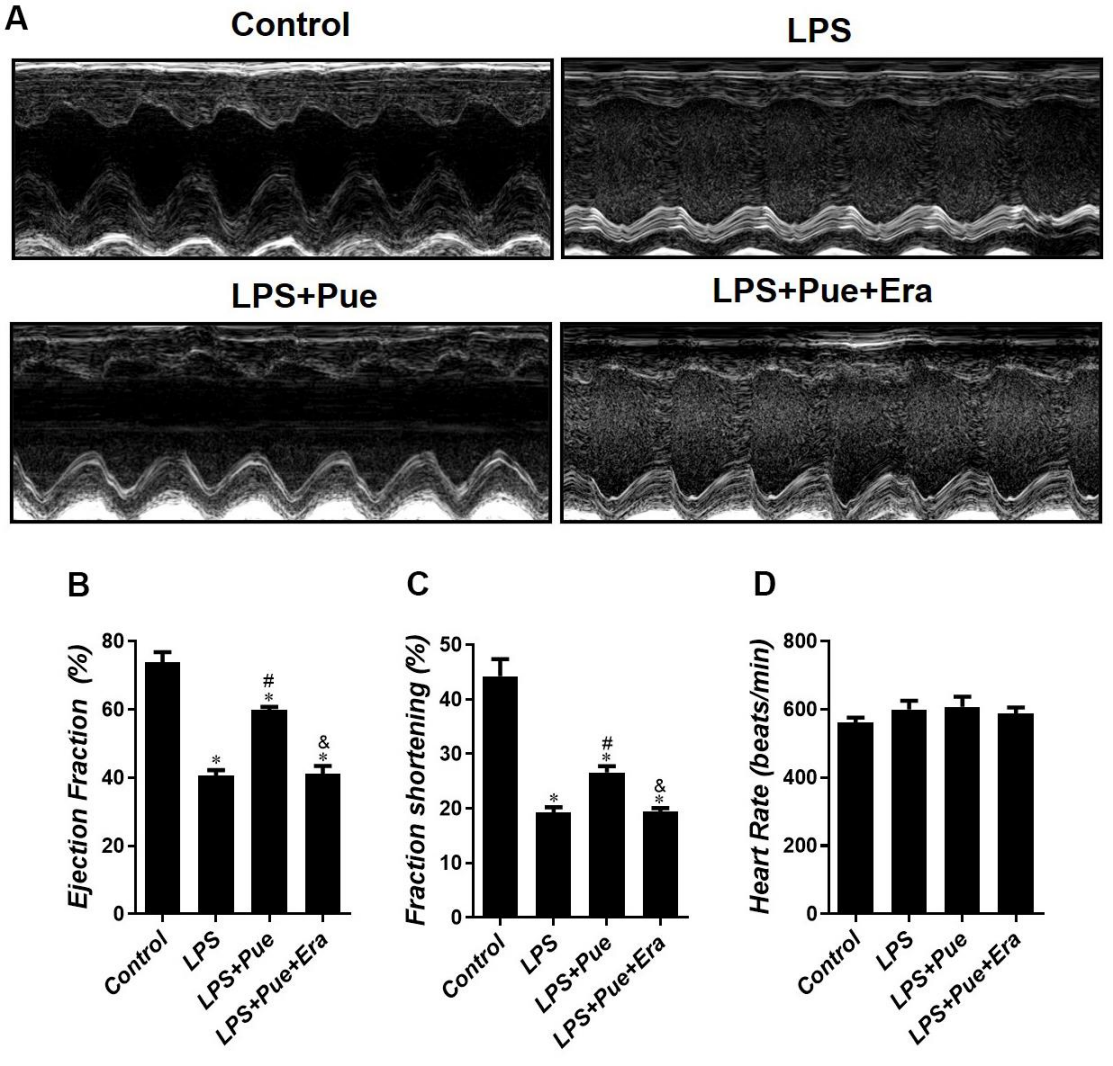
**Puerarin attenuates LPS-induced cardiac dysfunction in mice.** (**A**) Echocardiography was conducted to evaluate the changed in cardiac function in each group. Quantification of (**B**) ejection fraction (EF%), (**C**) fractional shortening (FS%) and (**D**) heart rate in all groups. Data represent the mean ± SD. *P < 0.05 vs Control group; #P < 0.05 vs. LPS group; &P < 0.05 vs. LPS+Pue group.

The level of CK-MB, LDH, and c-TnI in serum is the molecular indicator of myocardial injury. Pue was tested in the myocardium by measuring serum concentrations of CK-MB, LDH, and c-TnI after LPS, LPS + Pue, and LPS + Pue + Era. The results revealed that compared with the control group, the concentrations of CK-MB, LDH, and c-TnI in the LPS group were significantly (p < 0.05) increased. However, serum cardiac injury marker concentrations (CK-MB, LDH, and c-TnI) were significantly (p < 0.05) decreased after Pue pretreatment (Figure 1C–1E). Furthermore, serum levels of myocardial damage markers (CK-MB, LDH, and c-TnI) in rats predated with Pue + Era were significantly higher (p < 0.05) than those in LPS + Pue group (Figure 1C–1E). On echocardiography, LPS group rats had decreased ejection fraction (EF%) and fraction shortening (FS%) (p < 0.05) compared with the control group. In contrast, the systolic cardiac function of rats pretreated with Pue was significantly improved (p < 0.05) (Figure 2C, 2D). The data indicate that Puerarin protects against damage and dysfunction caused by LPS and that ERA inhibits Puerarin’s protective effects on the myocardium.

### Puerarin alleviates LPS-induced oxidative stress and inflammatory stress

**Figure 3:**
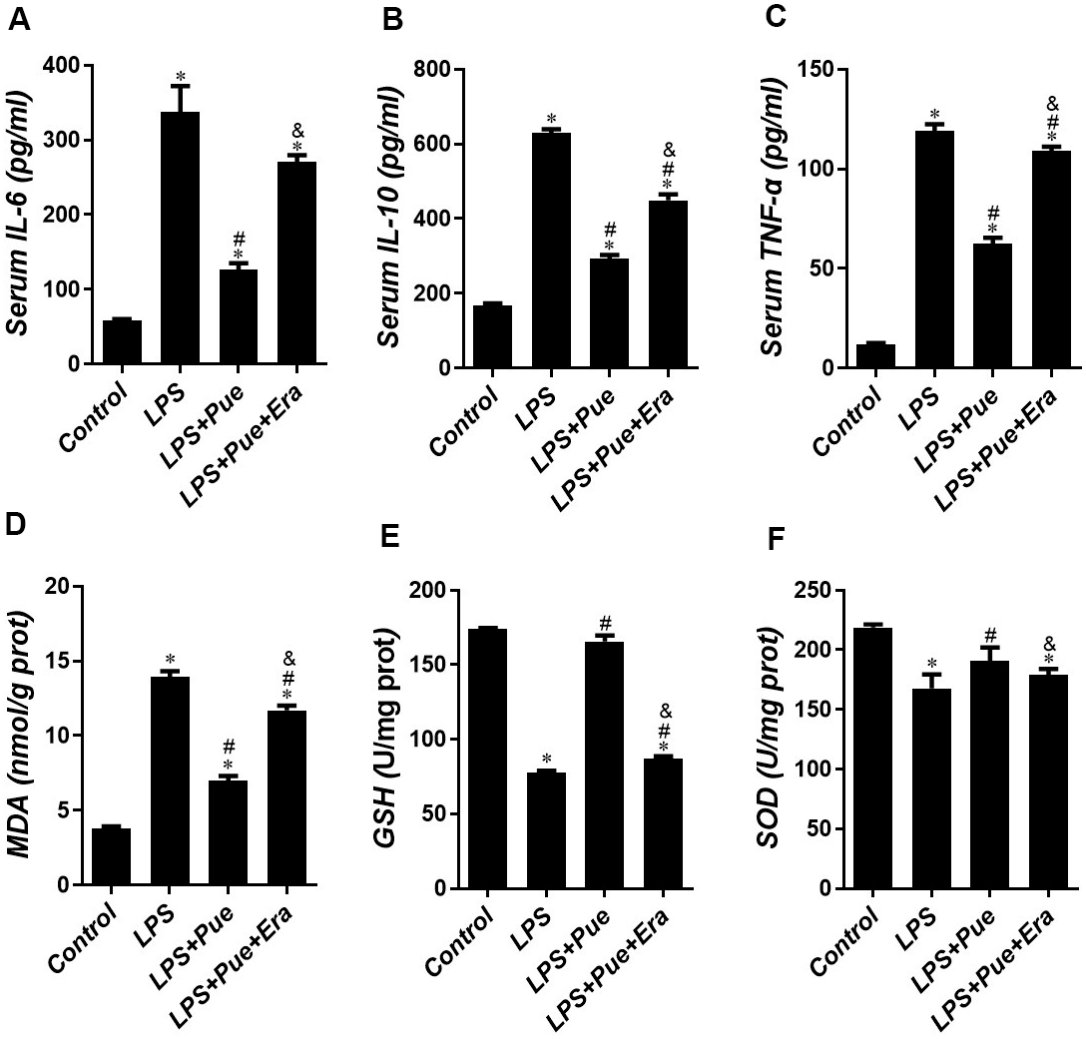
**Puerarin alleviates LPS-induced oxidative stress and inflammatory stress.** The concentrations of (**A**) IL-6, (**B**) IL-10 and (**C**) TNF-α, (**D**) MDA, (**E**) GSH and (**F**) SOD in serum of mice were determined by ELISA. Data represent the mean ± SD. *P < 0.05 vs Control group; #P < 0.05 vs. LPS group; &P < 0.05 vs. LPS+Pue group.

To investigate the protective effect of Puerarin on LPS-induced cardiac damage and dysfunction, we detected the expression levels of pro-inflammatory cytokines (TNF-α and IL-6), anti-inflammatory cytokines (IL-10), and oxidative stress indicators (MDA, GSH, and SOD) in rats of each group. The results demonstrated that compared with the control group, the levels of inflammatory cytokines (TNF-α, IL-6, and IL-10) and MDA in LPS group were significantly increased (p < 0.05) (Figure 3A–3D). Simultaneously, SOD and GSH values decreased significantly (p < 0.05) (Figure 3E, 3F). Moreover, the levels of inflammatory cytokines (TNF-α, IL-6, and IL-10) and MDA were significantly reduced (p < 0.05) after Pue pretreatment, and the values of SOD and GSH were enhanced considerably (p < 0.05) (Figure 3A–3F). Puerarin has been reported to inhibit LPS induced oxidative stress and inflammation.

### Puerarin inhibits apoptosis induced by LPS in rats via down-regulation of pro-apoptotic protein

**Figure 4:**
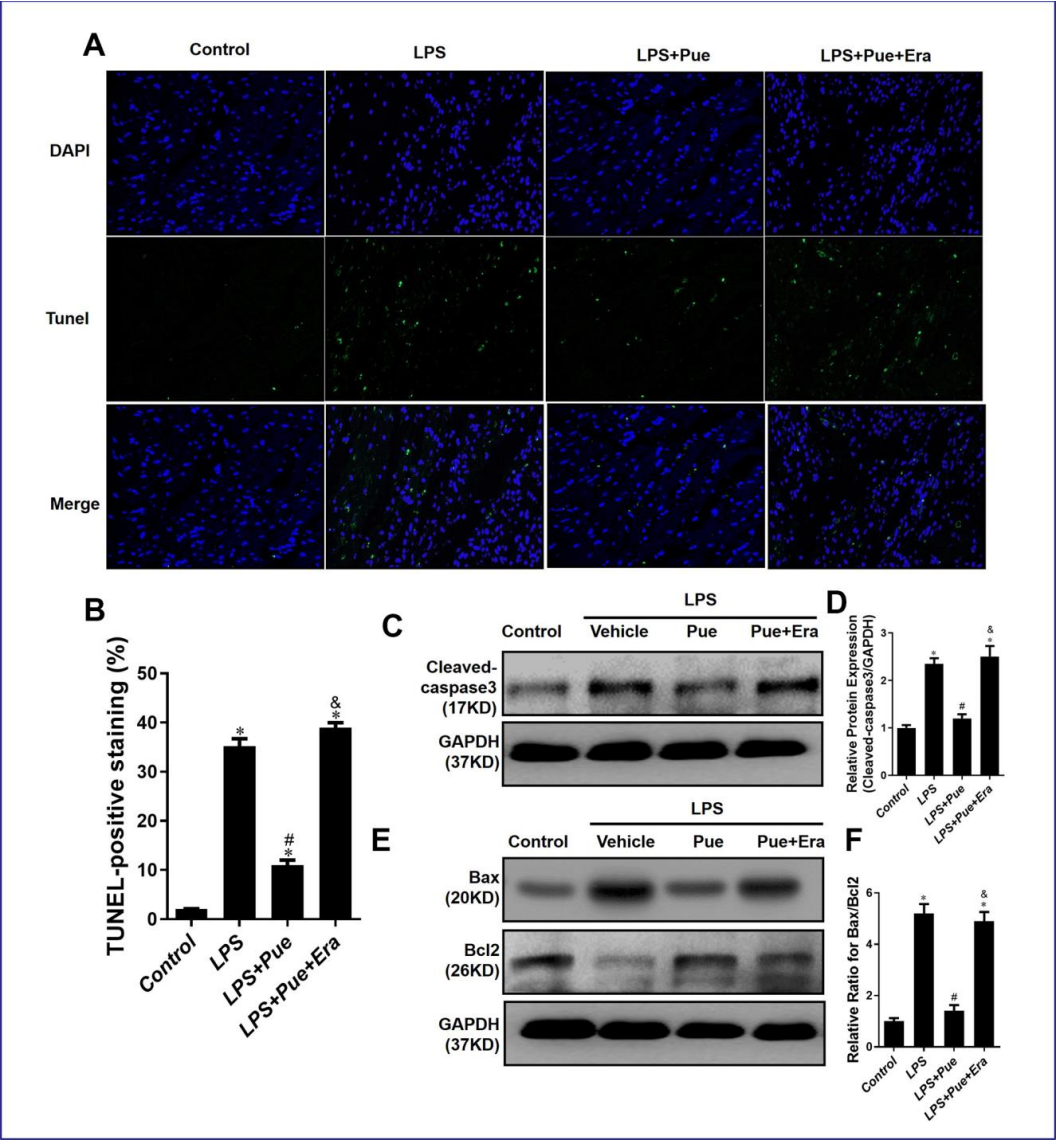
**Puerarin inhibits apoptosis of cardiomyocytes in septic mice.** (**A**, **B**) TUNEL staining was used to measure myocardial apoptosis in myocardial tissue sections. (**C**, **D**) Representative Western blot analysis and quantitative protein analysis of C-Caspase 3 expression in cardiac protein extract. (**E**, **F**) Representative Western blot analysis and quantitative protein analysis of Bax, and Bcl-2 expression in cardiac protein extract. Data represent the mean ± SD. *P < 0.05 vs Control group; #P < 0.05 vs. LPS group; &P < 0.05 vs. LPS+Pue group.

LPS can harm the myocardium by influencing the expression of apoptosis-related proteins. Tunel staining disclosed that compared to the control group, the number of Tunel positive nuclei increased significantly (p < 0.05) in LPS group, and decreased significantly (p < 0.05) after Pue pretreatment (Figure 4A, 4B). Furthermore, compared with LPS + Pue group, the number of Tunel positive nuclear staining was significantly higher (p < 0.05) in the group where rats were pretreated with Pue and Era. We then examined proteins associated with apoptosis (cleaved caspase-3, Bax, and Bcl-2) using western blot within all groups. The results indicated that compared to the control group, the expression level of cleaved caspase-3 and the Bax/Bcl-2 ratio increased significantly (p < 0.05) in LPS group, while the expression level of cleaved caspase-3 and the Bax/Bcl-2 ratio were significantly decreased (p < 0.05) after Pue pretreatment (Figure 4C–4F). Compared to LPS + Pue group, expression levels of cleaved caspase-3 and Bax/Bcl-2 ratio increased significantly (p < 0.05) in the group that added Era (Figure 4C–4F). It has been suggested that Puerarin may protect against LPS induced myocardial injury and cardiac dysfunction by inhibiting the expression of cleaved caspase-3 expression and reducing Bax/Bcl-2 ratio.

### Puerarin suppresses LPS-induced myocardial ferroptosis by up-regulation of AMPK phosphorylation

**Figure 5:**
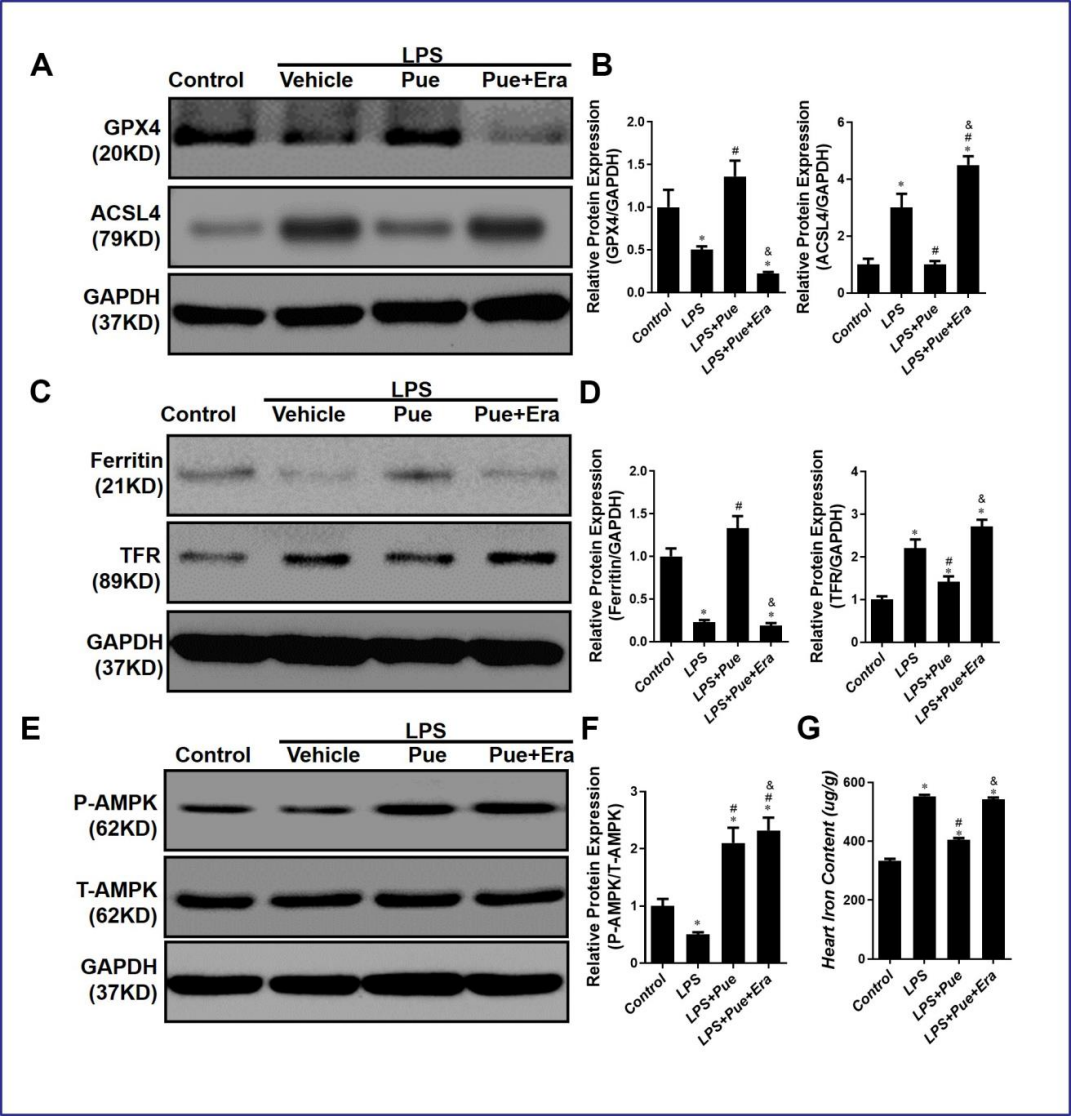
**Puerarin suppresses LPS-induced myocardial ferroptosis via up-regulation the phosphorylation of AMPK.** (**A**, **B**) Representative Western blot analysis and quantitative protein analysis of GPX4 and ACSL4 expression in cardiac protein extract. (**C**, **D**) Representative Western blot analysis and quantitative protein analysis of Ferritin and TFR expression in cardiac protein extract. (**E**, **F**) Representative Western blot analysis and quantitative protein analysis of P-AMPK expression in cardiac protein extract. (**G**) represents the amount of iron in the heart. Data represent the mean ± SD. *P < 0.05 vs Control group; #P < 0.05 vs. LPS group; &P < 0.05 vs. LPS+Pue group.

We hypothesized that Puerarin might inhibit AMPK-mediated ferroptosis signaling pathway, which we discovered from preliminary experiments and extensive literature review. Therefore, Western blot detected the protein associated with ferroptosis and AMPK. The experimental results revealed that, compared with the control group, the expression levels of ACSL4 and TFR in LPS group were significantly increased (p < 0.05), and the expression levels of GPX4 and Ferritin were decreased (p < 0.05). On the contrary, the expression levels of GPX4 and Ferritin significantly increased (p < 0.05), and the expression levels of ACSL4 and TFR decreased after pretreatment with Pue (Figure 5A–5D).

In addition, compared with LPS + Pue group, ACSL4 and TFR expressions in the group which added Era were significantly increased (p < 0.05), while GPX4 and Ferritin were decreased (p < 0.05) (Figure 5A–5D). Meanwhile, compared to the control group, P-AMPK/T-AMPK in the myocardial tissue of rats in LPS group decreased significantly (p < 0.05), while P-AMPK/T-AMPK in the myocardial tissue of rats pretreated with Pue increased (Figure 5E, 5F). Puerarin inhibits ferroptosis and modulates AMPK expression in rats, suggesting that it protects against LPS-induced myocardial injury.

Our conjecture was also confirmed when we measured the level of iron in the myocardium of rats in each group. Compared to the control group, the iron expression level in LPS group was significantly increased (p < 0.05). In contrast, the iron content in the myocardium was significantly decreased (p < 0.05) after pretreatment with Pue (Figure 5G). In addition, compared with LPS+Pue group, the iron content in the group which added Era was significantly increased (p < 0.05) (Figure 5G).

### AMPK inhibitor compound C abrogates ferroptosis, inhibiting effects of Puerarin

**Figure 6:**
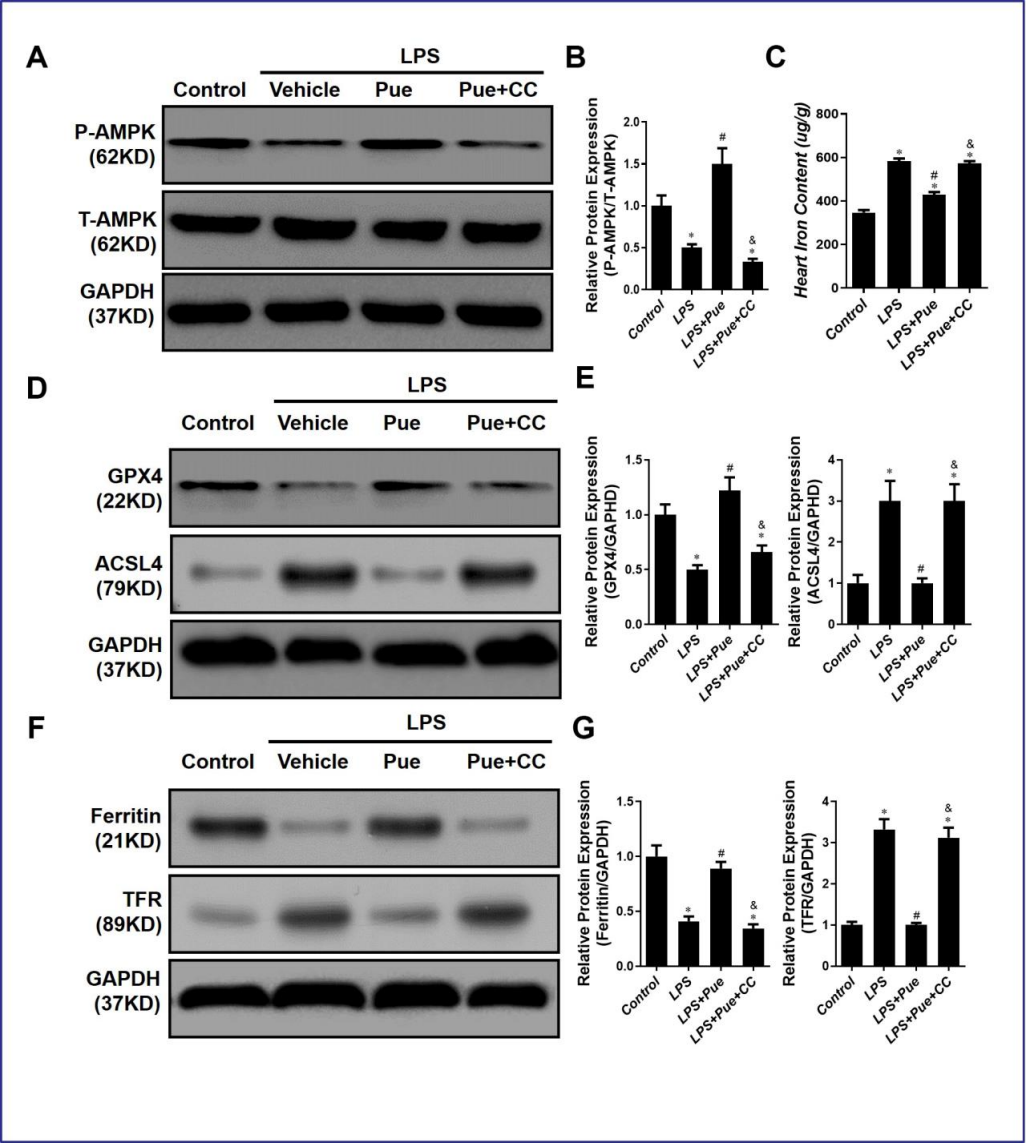
**AMPK inhibitor compound C abrogates ferroptosis inhibiting effects of Puerarin.** (**A**, **B**) Representative Western blot analysis and quantitative protein analysis of P-AMPK expression in cardiac protein extract. (**C**) Represents the amount of iron in the heart. (**D**, **E**) Representative Western blot analysis and quantitative protein analysis of GPX4 and ACSL4 expression in cardiac protein extract. (**F**, **G**) Representative Western blot analysis and quantitative protein analysis of Ferritin and TFR expression in cardiac protein extract. Data represent the mean ± SD. *P < 0.05 vs Control group; #P < 0.05 vs. LPS group; &P < 0.05 vs. LPS+Pue group.

We hypothesize that Puerarin inhibits ferroptosis by activating the AMPK signaling pathway, thereby protecting against LPS-induced myocardial injury and cardiac dysfunction. Using Western blots, we detected the protein associated with ferroptosis and AMPK in the rat’s second part of the experiment. The results revealed that, compared with the control group, ACSL4 and TFR expression levels in LPS group were significantly increased (p < 0.05), those of GPX4 and Ferritin were decreased (p < 0.05). However, GPX4 and Ferritin expression levels were significantly increased (p < 0.05), and those of ACSL4 and TFR were significantly decreased (p < 0.05) after Pue pretreatment (Figure 6D–6G). In addition, P-AMPK/T-AMPK was significantly reduced (p < 0.05) after the addition of AMPK inhibitor CC (Figure 6A, 6B). Meanwhile, compared to LPS + Pue group, GPX4 and ferritin decreased (p < 0.05), ACSL4 and TFR increased (p < 0.05) in LPS + Pue + CC group (Figure 6D–6G). This study suggests that Puerarin inhibits ferroptosis mediated by AMPK signaling pathway and that AMPK inhibitor CC can block this inhibition.

Similarly, we also detected iron in the myocardium of rats in each group, and the results further confirmed our hypothesis. Compared to the control group, the iron expression level in LPS group was significantly increased (p < 0.05). In contrast, the iron content in the myocardium was significantly decreased (p < 0.05) after Pue pretreatment (Figure 6C). In addition, compared with LPS+Pue group, the iron content in the group which added CC was significantly increased (p < 0.05) (Figure 6C).

## DISCUSSION

**Figure 7:**
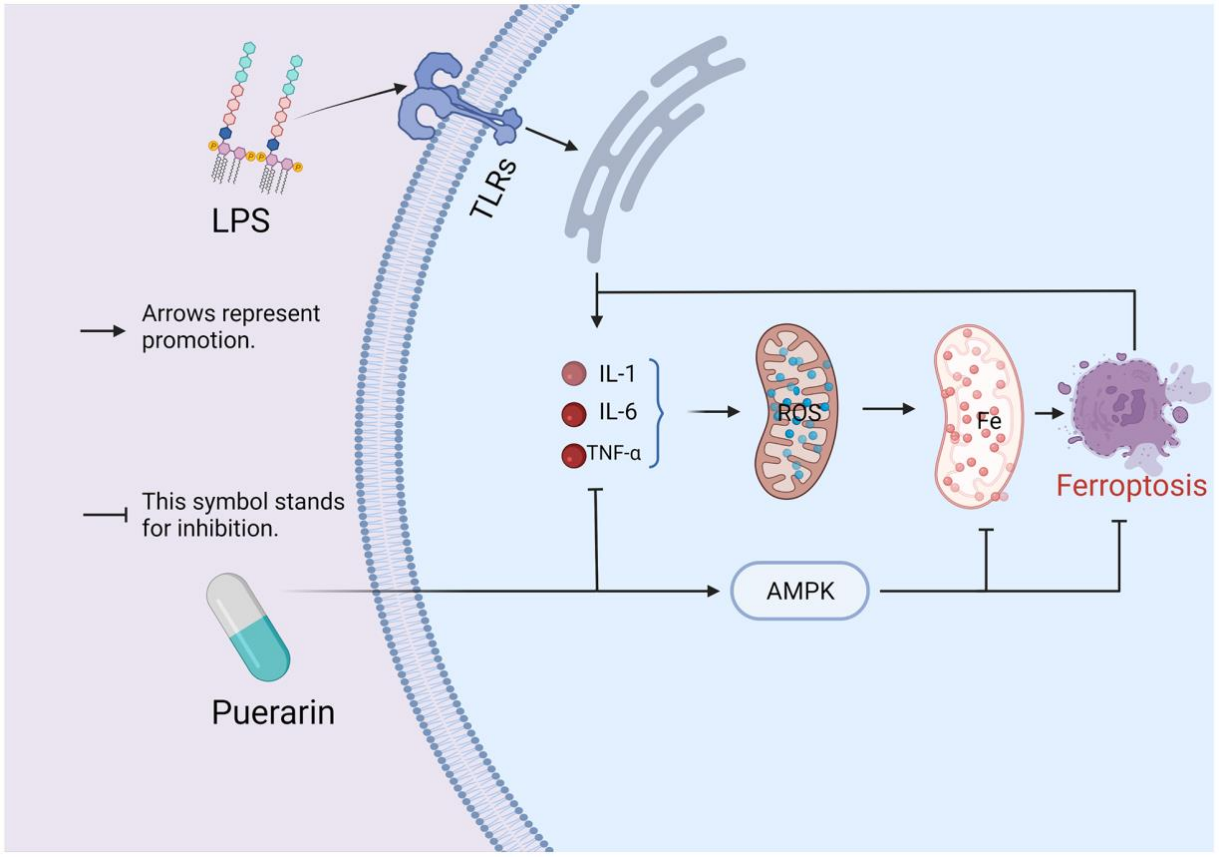
**A hypothesis on the mechanism of Puerarin on sepsis-induced myocardial injury: the combination of LPS and TLRs promotes the body to produce a large number of inflammatory cytokines TNF-α, IL-1β, and IL-6, which affect the coupling process of the oxidative respiratory chain in the mitochondria and causes the accumulation of ROS.** The accumulation of ROS can induce ferroptosis, which in turn leads to myocardial damage. However, in this study, the experimental results indicate that Puerarin may inhibit the occurrence of ferroptosis by activating AMPK, thereby exerting its cardioprotective effect.

Gram-negative bacteria produce LPS in their cell walls, one of the sepsis’s most critical pathogenic factors [5]. LPS-induced sepsis can cause the body to produce many inflammatory mediators (IL-1, IL-6, and TNF-α), resulting in a cascade of inflammatory responses that can cause extensive tissue and cell damage and damage to multiple organ structures. Injury and dysfunction eventually result in the patient’s death [20, 30, 31]. Experiments revealed that Puerarin, through its anti-inflammatory and antioxidant effects, can effectively improve LPS-induced myocardial injury, reduce the release of myocardial enzyme release, and maintain normal heart function. Western blot demonstrated that Puerarin inhibited the expressions of cleaved caspase-3, Bax, Bcl-2, ACSL4, and TFR, meanwhile up-regulating AMPK phosphorylation in heart tissues. Furthermore, we added compound C, an AMPK inhibitor, to LPS model rat, and subsequent experimental data suggested that Puerarin’s cardioprotective effect was possibly related to AMPK-mediated ferroptosis signaling pathway (Figure 7).

Sepsis is a systemic inflammatory response syndrome caused by infection [2] and is characterized by sepsis-induced cardiotoxicity [32]. Sepsis can cause myocardial injury and increase the concentration of myocardial damage markers (CK-MB and LDH) in mouse plasma [31]. In addition, it can lead to cardiac dysfunction manifested as a decline in ejection fraction (EF%) and fractional shortening (FS%) [31]. Studies have demonstrated that oxidative stress and inflammation caused by LPS through various signaling pathways are the main pathophysiological mechanisms of sepsis leading to myocardial damage and cardiac dysfunction [21]. LPS can up-regulate the expression of inflammatory cytokines through TLR4/NFκB signaling pathway to promote inflammation [33]. LPS can also enhance the oxidative stress of mouse myocardial tissue by inhibiting AMPK signaling pathway [34]. Researchers used many anti-inflammatory and antioxidant drugs to conduct experiments to determine the mechanism of LPS-induced myocardial injury. Leonurine inhibits oxidative stress induced by LPS by inhibiting NF-κB signaling pathway, thereby protecting the heart [35]. Salidroside inhibits the inflammatory response induced by LPS by inhibiting ROS-mediated PI3K/Akt/mTOR pathways, thus improving sepsis-induced cardiotoxicity [36]. Numerous studies have indicated that many traditional Chinese herbs have potent anti-inflammatory and antioxidant properties [12–15]. Due to various diseases, Puerarin can effectively reduce oxidative stress and inflammation in the myocardium [37, 38]. In addition, through our research, we demonstrate that Puerarin significantly reduced oxidative stress indicators (MDA, GSH, and SOD) and inflammatory cytokines (TNF-α, IL-6, and IL-10) in the serum of LPS model rats.

Puerarin is a monomer of traditional Chinese medicine derived from the Radix Pueraria, one of the most effective sources of isoflavones. The chemical name is 4, 7-dihydroxy-8-β-D-glucosyl isoflavone [16]. Puerarin is used to treat various diseases, and the concentration used for each disease varies [39, 40]. Puerarin has been revealed to successfully improve myocardial damage caused by coronary heart disease, diabetes, ischemia-reperfusion, hypoxia, and severe burns. Puerarin is notable for protecting against myocardial damage caused by various heart diseases through multiple mechanisms. Puerarin inhibits cardiomyocyte apoptosis by down-regulating the expression of apoptosis-related proteins cleaved-caspase 3 and Bax, increasing Bcl-2 expression, and increasing Bcl-2/Bax ratio [41].

Furthermore, we also confirmed this conclusion in our research. In a study of myocardial infarction, researchers found that Puerarin can protect against myocardial infarction by regulating PPAR-Υ/NF-κβ signaling pathway [42]. Puerarin inhibits inflammation by activating SIRT1/NF-κβ signaling pathway, improving myocardial ischemia-reperfusion injury [37].

Moreover, Puerarin can protect against myocardial injury caused by various diseases by modulating AMPK signaling pathway. In a study of myocardial ischemia-reperfusion injury, researchers found that Puerarin can reduce the expression of inflammatory cytokines IL-6, IL-1β, and TNF-α through AMPK/Akt/GSK-3β /Nrf2 signaling pathway to prevent myocardial ischemia-reperfusion injury [43]. Interestingly, researchers also conducted experiments with other drugs and discovered that AMPK could inhibit oxidative stress and inflammation induced by LPS after activation [44].

AMPK, an AMP-dependent protein kinase, is a key molecule that regulates bioenergy metabolism and a potential therapeutic target for various metabolic diseases [45]. AMPK is a heterotrimeric protein kinase composed of one catalytic subunit α and two regulatory subunits β and γ [46]. AMPK signaling pathway plays a vital role in the human body, and several substances influence the body through this signaling pathway [46]. Studies have found that luteolin can protect against LPS-induced myocardial injury by activating the AMPK signaling pathway [47], suggesting that activating the AMPK signaling pathway can effectively improve LPS-induced myocardial injury. In addition, other studies have indicated that activation of the AMPK signaling pathway can inhibit ferroptosis [26]. Ferroptosis is a non-apoptotic cell death that depends on iron and is regulated by lipid oxidation [23, 24].

Additionally, ferroptosis is implicated in organ damage and dysfunction caused by various diseases. Several scholars have suggested that GPX4, acyl-CoA synthase long-chain family member 4 (ACSL4), transferrin receptor (TFR), and ferritin (Ferritin) play a critical role in ferroptosis and can be used as ferroptosis occurrence detection metrics [48, 49]. As a result, our study also chose these four as ferroptosis monitoring indicators. Based on recent research, Puerarin can protect organs by directly inhibiting ferroptosis [29]. More importantly, ferroptosis is important in myocardial injury resulting from various heart diseases [50]. Ferroptosis inhibitors can effectively improve heart damage caused by various heart diseases. Researchers used ferrostatin-1 to treat H9c2 cells and found that ferrostatin-1 can inhibit the damage to cardiomyocytes caused by ischemia-reperfusion [51]. More interestingly, it has been recently confirmed that dexmedetomidine improves tissue and organ damage by inhibiting LPS-induced ferroptosis [25]. In addition, we discovered that ferroptosis activator Erastin could effectively abolish Puerarin’s protective effect on LPS-induced myocardial injury, confirming ferroptosis’s key role in myocardial injury. In echocardiography and HE stained sections, we found that Erastin combined with Puerarin exacerbated myocardial injury, the release of myocardial enzymes increased (CK-MB, LDH, and cTnI), while myocardial contractility decreased. By measuring the heart weights of the rats in each group, we discovered that compared to LPS group, HW/BW and HW/TL ratios of LPS+Pue group were significantly reduced (p < 0.05), demonstrating that Puerarin can alleviate LPS-induced myocardial edema (Figure 1B).

Furthermore, ELISA results show that Erastin in conjunction with Puerarin group increases the oxidation index MDA in cardiomyocytes while decreasing the antioxidant index GSH and SOD. We also observed that Erastin effectively abolished the anti-inflammatory effect of Puerarin. After adding Erastin, the expressions of IL-1, IL-6, and TNF-α in myocardial cells increased (Figure 3A–3C). In addition, Erastin can directly abolish the anti-apoptotic effect of Puerarin. The expression level of cleaved caspase-3 and Bax/Bcl-2 ratio in Erastin combined with Puerarin group were significantly higher than those of LPS+Pue group. Employing tissue-iron quantitation and Western blotting, we discovered that Puerarin could effectively reduce the iron content in the myocardium while promoting AMPK phosphorylation. Puerarin can effectively inhibit the expression of ferroptosis-promoting protein ferritin and ACSL4 and increase the expression of various anti-ferroptosis-related proteins (Figure 5). AMPK phosphorylation level in LPS+Pue group increased compared to that in LPS group. The expression of ferroptosis promoting proteins Ferritin and ACSL4 decreased, while the anti-ferroptosis proteins GPX4 and TFR increased (Figures 5, 6).

Furthermore, after AMPK inhibitor compound C inhibited AMPK activation, the regulatory effect of Puerarin on ferroptosis-related proteins was inhibited. Similarly, the iron content of CC-added group was significantly higher than that of LPS+Pue group (Figure 6). Our results indicate that Puerarin has an anti-ferroptosis effect, but this effect was blocked by pretreatment with AMPK inhibitor CC.

### Limitations

First, we only performed experiments *in vivo* using specific inhibitors to study the mechanisms. To elucidate further mechanisms, we must employ transgenic or knockout rat animal models or cells that overexpress AMPK and ferroptosis-related proteins. Moreover, this is only an animal experiment, and it must be proven in clinical trials before it can be used in humans.

## CONCLUSIONS

The present study demonstrated that Puerarin exerts cardioprotective effects against sepsis-induced cardiotoxicity. Puerarin exerts cardioprotective effects by attenuating inflammation and oxidative damage in myocardial tissues and suppressing apoptosis and ferroptosis in cardiomyocytes. The cardioprotective effects of Puerarin can be inhibited by inhibiting AMPK phosphorylation or promoting ferroptosis. Our results suggest that targeting AMPK or ferroptosis key proteins could be vital for future prevention and treatment of LPS-induced myocardial injury. More research is required to investigate the clinical application of Puerarin.

## MATERIALS AND METHODS

### Animals and materials

48 healthy male Sprague-Dawley rats (aged 8-10 weeks) were purchased from Model Animal Research Center. Puerarin was obtained from Sigma-Aldrich (P5555). Lipopolysaccharide (LPS, ST1470) and One Step TUNEL Apoptosis Assay Kit (C1088) were purchased from Beyotime Biotechnology. The following antibodies were used in this study: anti-cleaved caspase3 (Cell Signaling Technology, 9661), anti-Bax (Cell Signaling Technology, 2772), anti-Bcl2 (Cell Signaling Technology, 3498), anti-GPX4 (Cell Signaling Technology, 52455), anti-ACSL4 (Santa Cruz Biotechnology, sc-365230), anti-Ferritin (Abcam, ab75973), anti-TFR (Abcam, ab84036), anti-P-AMPK (Cell Signaling Technology, 50081), anti-T-AMPK (Cell Signaling Technology, 5832), and anti-GADPH (Beyotime Biotechnology, AF0006). Then enzyme-linked immunosorbent assay (ELISA) kit for the detection of IL-6 (H007), IL-10 (H009) and TNF-α (H052) were purchased from Nanjing Jiancheng Bioengineering Institute. In addition, oxidative stress indicators (MDA, GSH, and SOD) were detected by the ELISA kit from Nanjing Jiancheng Bioengineering Institute.

### Animal treatment

The rats were raised in the Experimental Animal Center of Nanchang University. The experimental center adopted a standard feeding environment (20-25° C, 50 ± 5% relative humidity, 12-hour day-night cycle light), and were fed with standard fresh water for at least one week before the experiment. All animal experiments in this study were performed with the approval of the Animal Care and Use Committee of the Second Affiliated Hospital of Nanchang University.

After adaptation to the housed environment for 1 week, rats were then randomized into these groups: Control group, LPS group, LPS + Pue group, LPS + Pue + Era (Erastin, ferroptosis activator) group and LPS + Pue + CC (Compound C, AMPK inhibitor) group. All groups were intraperitoneally injected with LPS 10mg/kg to establish LPS model except the Control group. The Control group was intraperitoneally injected with the same amount of normal saline. The LPS + Pue group, LPS + Pue + Era group and LPS + Pue + CC group were respectively intraperitoneally injected with Pue 100mg/kg, Pue 100mg/kg + Era 10umol/kg and Pue 100mg/kg + CC 0.2mg/kg half an hour after LPS injection.

### Hematoxylin and eosin (H&E) staining

First of all, rat myocardial tissue was fixed in 10% paraformaldehyde for 24 hours, embedded with paraffin. The embedded tissues were then sliced into sections of 3-5 μm, dewaxed with xylene twice for 5 min each and hydrated by ethanol. Then they were dyed with sappanwood acid for 10 minutes and rinse with running water. After ten seconds of 1% HCL ethanol differentiation, dyed it with 5% eosin for 1-3 minutes. And then dehydrated with gradient ethanol and dewaxed with xylene. Finally, air-dried slices were sealed with neutral resin and observed under a Leica IX71 microscope.

### Determination of cardiac enzymes and cardiac weight analysis

First, mice were anesthetized by continuous administration of isoflurane at 1.5%. The anaesthetized rats were sacrificed to obtain their whole blood. Then the concentrations of CK-MB, LDH and c-TnI in the serum of rat were measured by ELISA, and all the steps were performed in strict accordance with the ELISA kits instructions. In addition, after the rats were sacrificed, the hearts were promptly harvested and weighed. Relative HW/BW and HW/TL ratios were calculated.

### Echocardiography

Mice were anesthetized by continuous administration of isoflurane at 1.5%, fastened to a heating pad in the supine position and the chest hair was shaved. We used the two-dimensional echocardiography equipped with a 35-MHz linear transducer to detect the cardiac systolic function indicators of the rat, including the left ventricular end-systolic diameter (LVSD), the left ventricular end-diastolic diameter (LVDD), left ventricular posterior wall thickness (LVPWT), and heart rate. And we measured LV end systolic and diastolic diameter (LVEDS, LVEDD), LV end systolic and diastolic posterior wall thickness (LVPWS, LVPWDD). According to the LVEDS and LVEDD data, the ejection fraction (EF%) and fraction shortening (FS%) were gained via automatic calculation.

### Enzyme-linked immunosorbent assay (ELISA)

The levels of proinflammatory cytokines (IL-1, TNF-α, and IL-6), anti-inflammatory cytokine (IL-10), and oxidative stress indicators (MDA, GSH, and SOD) in each group were measured by ELISA, and all the steps were performed in strict accordance with the ELISA kits instructions.

### Terminal deoxynucleotidyl transferase dUTP nick end labeling (Tunel) staining

The myocardial tissue of rat was extracted, paraffin-embedded, sliced, dewaxed and rehydrated, and then Tunel Kit was used to stain the myocardial tissue. Finally, all slices were observed and images were collected.

### Western blot analysis and tissue-iron quantitation

Myocardial tissue was cut from rats in each group, and then homogenized in protein lysis buffer. After centrifugation for 20 minutes (4° C, 12000 g), supernatant was taken and quantified by BCA colorimetric method. The protein was separated by electrophoresis on SDS-PAGE glue and transferred to the polyvinylidene fluoride membranes. The protein was sealed with 5% skim milk. The desired bands were then placed in the corresponding diluted 1000 times primary antibodies (Bax, Bcl2, GPX4, ACSL4, Ferritin, TFR, P-AMPK, T-AMPK, cleaved caspase3, and GADPH) and incubated overnight. After washing the film with PBS for 3 times, the membranes were incubated with a secondary antibody for 1.5h, and then washed with PBS for 3 times. Subsequently, bands were analysed with an ECL detection kit.

Myocardial tissue was cut from rats in each group, and then tissue homogenates are treated with hydrochloric acid and trichloroacetic acid and heated at 95° C. Following centrifugation, iron in the supernatant is reacted with ferrozine in the presence of the reducing agent thioglycolic acid, and the complex is quantified by spectrophotometry.

### Statistical methods

The datum is described as the mean ± standard deviation of at least three independent experiments. Statistical software SPSS 20.0 was used to evaluate the statistical significance of the differences between groups by one-way ANOVA. P values < 0.05 were considered statistically significant.

## References

[1] Sun X, Dai Y, Tan G, Liu Y, Li N. Integration Analysis of m^6^A-SNPs and eQTLs Associated With Sepsis Reveals Platelet Degranulation and Staphylococcus aureus Infection are Mediated by m^6^A mRNA Methylation. Front Genet. 2020; 11:7. 10.3389/fgene.2020.00007 32174955 PMC7054457

[2] Simpson BW, Trent MS. Pushing the envelope: LPS modifications and their consequences. Nat Rev Microbiol. 2019; 17:403–16. 10.1038/s41579-019-0201-x 31142822 PMC6913091

[3] Deutschman CS, Tracey KJ. Sepsis: current dogma and new perspectives. Immunity. 2014; 40:463–75. 10.1016/j.immuni.2014.04.001 24745331

[4] Dellinger RP, Levy MM, Rhodes A, Annane D, Gerlach H, Opal SM, Sevransky JE, Sprung CL, Douglas IS, Jaeschke R, Osborn TM, Nunnally ME, Townsend SR, et al, and Surviving Sepsis Campaign Guidelines Committee including The Pediatric Subgroup. Surviving Sepsis Campaign: international guidelines for management of severe sepsis and septic shock, 2012. Intensive Care Med. 2013; 39:165–228. 10.1007/s00134-012-2769-8 23361625 PMC7095153

[5] Lu X, Wang J, Chen X, Jiang Y, Pan ZK. Rolipram Protects Mice from Gram-negative Bacterium Escherichia coli-induced Inflammation and Septic Shock. Sci Rep. 2020; 10:175. 10.1038/s41598-019-56899-6 31932743 PMC6957694

[6] Meares GP, Qin H, Liu Y, Holdbrooks AT, Benveniste EN. AMP-activated protein kinase restricts IFN-γ signaling. J Immunol. 2013; 190:372–80. 10.4049/jimmunol.1202390 23180823 PMC3735359

[7] Hochstadt A, Meroz Y, Landesberg G. Myocardial dysfunction in severe sepsis and septic shock: more questions than answers? J Cardiothorac Vasc Anesth. 2011; 25:526–35. 10.1053/j.jvca.2010.11.026 21296000

[8] Frencken JF, Donker DW, Spitoni C, Koster-Brouwer ME, Soliman IW, Ong DS, Horn J, van der Poll T, van Klei WA, Bonten MJ, Cremer OL, de Beer FM, Bos LD, et al. Myocardial Injury in Patients With Sepsis and Its Association With Long-Term Outcome. Circ Cardiovasc Qual Outcomes. 2018; 11:e004040. 10.1161/CIRCOUTCOMES.117.004040 29378734

[9] Li M, Gou Y, Yu H, Ji T, Li Y, Qin L, Sun W. Mechanism of Metformin on LPS-Induced Bacterial Myocarditis. Dose Response. 2019; 17:1559325819847409. 10.1177/1559325819847409 31205455 PMC6537499

[10] Zhao P, Zhang L, Gao L, Ding Q, Yang Q, Kuai J. Ulinastatin attenuates lipopolysaccharide-induced cardiac dysfunction by inhibiting inflammation and regulating autophagy. Exp Ther Med. 2020; 20:1064–72. 10.3892/etm.2020.8755 32765659 PMC7388552

[11] Chen H, Liu Q, Liu X, Jin J. Berberine attenuates septic cardiomyopathy by inhibiting TLR4/NF-κB signalling in rats. Pharm Biol. 2021; 59:121–8. 10.1080/13880209.2021.1877736 33539718 PMC8871679

[12] Zhang Y, Zhao Z, Tang E, Hu Y, Mao J. Additional traditional Chinese medicine on gastrointestinal dysfunction in patients with sepsis: A systematic review and meta-analysis. Pak J Pharm Sci. 2016 (Suppl 2); 29:663–9. 27113305

[13] Liu D, Tang S, Gan L, Cui W. Renal-Protective Effects and Potential Mechanisms of Traditional Chinese Medicine after Ischemia-Reperfusion Injury. Evid Based Complement Alternat Med. 2021; 2021:5579327. 10.1155/2021/5579327 33680054 PMC7910071

[14] Huang P, Zhao GZ, Chen YS, Ha YX, Zhang R, Hu J, Feng S, Guo YH, He SS, Liao X, Xie YM, Zhang JH, Zhang BL, Li B, Liu QQ. [Interpretation and prospect of clinical practice guideline on traditional Chinese medicine therapy alone or combined with antibiotics for sepsis]. Zhongguo Zhong Yao Za Zhi. 2018; 43:4782–5. 10.19540/j.cnki.cjcmm.20181009.005 30717519

[15] Park SY, Jin ML, Ko MJ, Park G, Choi YW. Anti-neuroinflammatory Effect of Emodin in LPS-Stimulated Microglia: Involvement of AMPK/Nrf2 Activation. Neurochem Res. 2016; 41:2981–92. 10.1007/s11064-016-2018-6 27538959

[16] Zhou YX, Zhang H, Peng C. Puerarin: a review of pharmacological effects. Phytother Res. 2014; 28:961–75. 10.1002/ptr.5083 24339367

[17] Wang L, Liang Q, Lin A, Chen X, Wu Y, Zhang B, Zhang Y, Min H, Wen Y, Song S, Gao Q. Puerarin Increases Survival and Protects Against Organ Injury by Suppressing NF-κB/JNK Signaling in Experimental Sepsis. Front Pharmacol. 2020; 11:560. 10.3389/fphar.2020.00560 32457606 PMC7221141

[18] Yuan Y, Zhou H, Wu QQ, Li FF, Bian ZY, Deng W, Zhou MQ, Tang QZ. Puerarin attenuates the inflammatory response and apoptosis in LPS-stimulated cardiomyocytes. Exp Ther Med. 2016; 11:415–20. 10.3892/etm.2015.2910 26893624 PMC4734177

[19] Shaojun Z, Yanyan X, Jian C, Xia Z, Qiang F, Saiping J. Effects of puerarin on lipopolysaccharide-induced myocardial dysfunction in isolated rat hearts. Pak J Pharm Sci. 2017; 30:1195–202. 29039314

[20] Qiu Z, He Y, Ming H, Lei S, Leng Y, Xia ZY. Lipopolysaccharide (LPS) Aggravates High Glucose- and Hypoxia/Reoxygenation-Induced Injury through Activating ROS-Dependent NLRP3 Inflammasome-Mediated Pyroptosis in H9C2 Cardiomyocytes. J Diabetes Res. 2019; 2019:8151836. 10.1155/2019/8151836 30911553 PMC6398034

[21] Xu J, Lin C, Wang T, Zhang P, Liu Z, Lu C. Ergosterol Attenuates LPS-Induced Myocardial Injury by Modulating Oxidative Stress and Apoptosis in Rats. Cell Physiol Biochem. 2018; 48:583–92. 10.1159/000491887 30021198

[22] Li N, Wang W, Zhou H, Wu Q, Duan M, Liu C, Wu H, Deng W, Shen D, Tang Q. Ferritinophagy-mediated ferroptosis is involved in sepsis-induced cardiac injury. Free Radic Biol Med. 2020; 160:303–18. 10.1016/j.freeradbiomed.2020.08.009 32846217

[23] Dixon SJ, Lemberg KM, Lamprecht MR, Skouta R, Zaitsev EM, Gleason CE, Patel DN, Bauer AJ, Cantley AM, Yang WS, Morrison B 3rd, Stockwell BR. Ferroptosis: an iron-dependent form of nonapoptotic cell death. Cell. 2012; 149:1060–72. 10.1016/j.cell.2012.03.042 22632970 PMC3367386

[24] Su LJ, Zhang JH, Gomez H, Murugan R, Hong X, Xu D, Jiang F, Peng ZY. Reactive Oxygen Species-Induced Lipid Peroxidation in Apoptosis, Autophagy, and Ferroptosis. Oxid Med Cell Longev. 2019; 2019:5080843. 10.1155/2019/5080843 31737171 PMC6815535

[25] Wang C, Yuan W, Hu A, Lin J, Xia Z, Yang CF, Li Y, Zhang Z. Dexmedetomidine alleviated sepsis-induced myocardial ferroptosis and septic heart injury. Mol Med Rep. 2020; 22:175–84. 10.3892/mmr.2020.11114 32377745 PMC7248514

[26] Lee H, Zandkarimi F, Zhang Y, Meena JK, Kim J, Zhuang L, Tyagi S, Ma L, Westbrook TF, Steinberg GR, Nakada D, Stockwell BR, Gan B. Energy-stress-mediated AMPK activation inhibits ferroptosis. Nat Cell Biol. 2020; 22:225–34. 10.1038/s41556-020-0461-8 32029897 PMC7008777

[27] Liu B, Wu Z, Li Y, Ou C, Huang Z, Zhang J, Liu P, Luo C, Chen M. Puerarin prevents cardiac hypertrophy induced by pressure overload through activation of autophagy. Biochem Biophys Res Commun. 2015; 464:908–15. 10.1016/j.bbrc.2015.07.065 26188094

[28] Tang H, Song X, Ling Y, Wang X, Yang P, Luo T, Chen A. Puerarin attenuates myocardial hypoxia/reoxygenation injury by inhibiting autophagy via the Akt signaling pathway. Mol Med Rep. 2017; 15:3747–54. 10.3892/mmr.2017.6424 28393209

[29] Liu B, Zhao C, Li H, Chen X, Ding Y, Xu S. Puerarin protects against heart failure induced by pressure overload through mitigation of ferroptosis. Biochem Biophys Res Commun. 2018; 497:233–40. 10.1016/j.bbrc.2018.02.061 29427658

[30] de Pádua Lúcio K, Rabelo ACS, Araújo CM, Brandão GC, de Souza GHB, da Silva RG, de Souza DMS, Talvani A, Bezerra FS, Cruz Calsavara AJ, Costa DC. Anti-Inflammatory and Antioxidant Properties of Black Mulberry (Morus nigra L.) in a Model of LPS-Induced Sepsis. Oxid Med Cell Longev. 2018; 2018:5048031. 10.1155/2018/5048031 30524657 PMC6247390

[31] Hu H, Fu Y, Li M, Xia H, Liu Y, Sun X, Hu Y, Song F, Cheng X, Li P, Wu Y. Interleukin-35 pretreatment attenuates lipopolysaccharide-induced heart injury by inhibition of inflammation, apoptosis and fibrotic reactions. Int Immunopharmacol. 2020; 86:106725. 10.1016/j.intimp.2020.106725 32679538

[32] Merx MW, Weber C. Sepsis and the heart. Circulation. 2007; 116:793–802. 10.1161/CIRCULATIONAHA.106.678359 17698745

[33] Badshah H, Ali T, Kim MO. Osmotin attenuates LPS-induced neuroinflammation and memory impairments via the TLR4/NFκB signaling pathway. Sci Rep. 2016; 6:24493. 10.1038/srep24493 27093924 PMC4837357

[34] Hwang HJ, Kim JW, Chung HS, Seo JA, Kim SG, Kim NH, Choi KM, Baik SH, Yoo HJ. Knockdown of Sestrin2 Increases Lipopolysaccharide-Induced Oxidative Stress, Apoptosis, and Fibrotic Reactions in H9c2 Cells and Heart Tissues of Mice via an AMPK-Dependent Mechanism. Mediators Inflamm. 2018; 2018:6209140. 10.1155/2018/6209140 30116150 PMC6079459

[35] Wang R, Li D, Ouyang J, Tian X, Zhao Y, Peng X, Li S, Yu G, Yang J. Leonurine alleviates LPS-induced myocarditis through suppressing the NF-кB signaling pathway. Toxicology. 2019; 422:1–13. 10.1016/j.tox.2019.04.011 31005592

[36] Chen L, Liu P, Feng X, Ma C. Salidroside suppressing LPS-induced myocardial injury by inhibiting ROS-mediated PI3K/Akt/mTOR pathway *in vitro* and *in vivo*. J Cell Mol Med. 2017; 21:3178–89. 10.1111/jcmm.12871 28905500 PMC5706507

[37] Wang ZK, Chen RR, Li JH, Chen JY, Li W, Niu XL, Wang FF, Wang J, Yang JX. Puerarin protects against myocardial ischemia/reperfusion injury by inhibiting inflammation and the NLRP3 inflammasome: The role of the SIRT1/NF-κB pathway. Int Immunopharmacol. 2020; 89:107086. 10.1016/j.intimp.2020.107086 33068868

[38] Liu J, Liu J, Bai M, Wang H. Protective effect of puerarin against burn-induced heart injury in rats. Exp Ther Med. 2020; 20:275–82. 10.3892/etm.2020.8696 32536996 PMC7282049

[39] Hou Q, Ao X, Li G, Zhang Y. [Puerarin combined with avandia for diabetic nephropathy]. Zhong Nan Da Xue Xue Bao Yi Xue Ban. 2012; 37:73–7. 10.3969/j.issn.1672-7347.2012.01.013 22349389

[40] Li W, Hu H, Zou G, Ma Z, Liu J, Li F. Therapeutic effects of puerarin on polycystic ovary syndrome: A randomized trial in Chinese women. Medicine (Baltimore). 2021; 100:e26049. 10.1097/MD.0000000000026049 34032731 PMC8154455

[41] Xu HX, Pan W, Qian JF, Liu F, Dong HQ, Liu QJ. MicroRNA-21 contributes to the puerarin-induced cardioprotection via suppression of apoptosis and oxidative stress in a cell model of ischemia/reperfusion injury. Mol Med Rep. 2019; 20:719–27. 10.3892/mmr.2019.10266 31115556

[42] Li X, Yuan T, Chen D, Chen Y, Sun S, Wang D, Fang L, Lu Y, Du G. Cardioprotective Effects of Puerarin-V on Isoproterenol-Induced Myocardial Infarction Mice Is Associated with Regulation of PPAR-Υ/NF-κB Pathway. Molecules. 2018; 23:3322. 10.3390/molecules23123322 30558188 PMC6321361

[43] Li ZF, Wang W, Jiang L, Liu X, Wu YH. Puerarin protects against myocardial ischemia/reperfusion injury via the AMPK/Akt/GSK-3β/Nrf2 signaling pathway. Int J Clin Exp Med. 2018; 11:4548–54. https://e-century.us/files/ijcem/11/5/ijcem0067705.pdf

[44] Kim N, Lertnimitphun P, Jiang Y, Tan H, Zhou H, Lu Y, Xu H. Andrographolide inhibits inflammatory responses in LPS-stimulated macrophages and murine acute colitis through activating AMPK. Biochem Pharmacol. 2019; 170:113646. 10.1016/j.bcp.2019.113646 31545974

[45] Carling D. AMPK signalling in health and disease. Curr Opin Cell Biol. 2017; 45:31–7. 10.1016/j.ceb.2017.01.005 28232179

[46] Yan Y, Zhou XE, Xu HE, Melcher K. Structure and Physiological Regulation of AMPK. Int J Mol Sci. 2018; 19:3534. 10.3390/ijms19113534 30423971 PMC6274893

[47] Wu B, Song H, Fan M, You F, Zhang L, Luo J, Li J, Wang L, Li C, Yuan M. Luteolin attenuates sepsis-induced myocardial injury by enhancing autophagy in mice. Int J Mol Med. 2020; 45:1477–87. 10.3892/ijmm.2020.4536 32323750 PMC7138288

[48] Xie Y, Hou W, Song X, Yu Y, Huang J, Sun X, Kang R, Tang D. Ferroptosis: process and function. Cell Death Differ. 2016; 23:369–79. 10.1038/cdd.2015.158 26794443 PMC5072448

[49] Park E, Chung SW. ROS-mediated autophagy increases intracellular iron levels and ferroptosis by ferritin and transferrin receptor regulation. Cell Death Dis. 2019; 10:822. 10.1038/s41419-019-2064-5 31659150 PMC6817894

[50] Fang X, Wang H, Han D, Xie E, Yang X, Wei J, Gu S, Gao F, Zhu N, Yin X, Cheng Q, Zhang P, Dai W, et al. Ferroptosis as a target for protection against cardiomyopathy. Proc Natl Acad Sci USA. 2019; 116:2672–80. 10.1073/pnas.1821022116 30692261 PMC6377499

[51] Son E, Lee D, Woo CW, Kim YH. The optimal model of reperfusion injury *in vitro* using H9c2 transformed cardiac myoblasts. Korean J Physiol Pharmacol. 2020; 24:173–83. 10.4196/kjpp.2020.24.2.173 32140041 PMC7043999

